# EFFECTS OF POSTRETIREMENT ACTIVITIES ON RETIREES’ WELL-BEING: THE MODERATING ROLE OF CHILDLESSNESS

**DOI:** 10.1093/geroni/igae098.2089

**Published:** 2024-12-31

**Authors:** Gloria Lin, Edwin Ka Hung Chung, Dannii Y Yeung, Alvin Ho, Amy Ng

**Affiliations:** City University of Hong Kong, Hong Kong, China (People’s Republic); City University of Hong Kong, Hong Kong, China (People’s Republic); City University of Hong Kong, Hong Kong, China (People’s Republic); City University of Hong Kong, Hong Kong, China (People’s Republic); City University of Hong Kong, Hong Kong, China (People’s Republic)

## Abstract

According to role theory, retirees’ work roles are likely diminished upon retirement, but their family and community roles are strengthened. Such pattern however may be different among childless retirees as their family roles are comparatively fewer than retirees with children. Postretirement activities, such as volunteering (Filges et al, 2020) and postretirement employment (Wang, 2007), have been shown to contribute to retirees’ well-being as they compensate for the absence of important roles after retirement. Thereby, this cross-sectional study aimed to investigate whether the relationship between postretirement activities and well-being would be moderated by childlessness. Retirees’ number of children, participation in postretirement activities (volunteering, postretirement employment, both activities, or none), life satisfaction, positive affect, depressive symptoms, and cognitive functioning were measured among 942 older Hong Kong Chinese retirees aged 65 and above (Mage = 71.76, SD = 5.28). The results of two-way ANCOVAs demonstrated significant main effects of postretirement activities and childlessness on most of well-being outcomes. A significant interaction effect of postretirement activities and childlessness on depressive symptoms was found, even after controlling for demographic variables. Specifically, childless retirees participated in volunteering (adj. Mdiff = –2.33, p =.001), postretirement employment (adj. Mdiff = –3.56, p =.001), or both activities (adj. Mdiff = –2.77, p =.010) showed fewer depressive symptoms than those with children. Our findings suggest that beneficial effects of postretirement activities are more salient among childless retirees than those with children, implying that participation in postretirement activities would be a protective factor in maintaining well-being after retirement.

